# Prognostic and Predictive Factors in Elderly Patients With Glioblastoma: A Single-Center Retrospective Study

**DOI:** 10.3389/fnagi.2021.777962

**Published:** 2022-01-31

**Authors:** Jinghui Liu, Chen Li, Yuan Wang, Peigang Ji, Shaochun Guo, Yulong Zhai, Na Wang, Miao Lou, Meng Xu, Min Chao, Yang Jiao, Wenjian Zhao, Fuqiang Feng, Yan Qu, Shunnan Ge, Liang Wang

**Affiliations:** ^1^Department of Neurosurgery, Tangdu Hospital, The Fourth Military Medical University, Xi’an, China; ^2^Department of Otolaryngology Head and Neck Surgery, The Second Affiliated Hospital of Xi’an Jiaotong University, Xi’an, China; ^3^Department of Neurosurgery, The Second Hospital of Shanxi Medical University, Taiyuan, China

**Keywords:** elderly, glioblastoma, Karnofsky performance scale score, prognosis (carcinoma), extent of resection (EOR)

## Abstract

Glioblastoma (GBM) is the most common primary malignant intracranial tumor and the median age at diagnosis is 65 years. However, elderly patients are usually excluded from clinical studies and age is considered as an independent negative prognostic factor for patients with GBM. Therefore, the best treatment method for GBM in elderly patients has remained controversial. Elderly GBM patients (≥ 60 years old) treated between January 2015 and December 2019 were enrolled in this study. Medical records were reviewed retrospectively, and clinicopathological characteristics, treatments, and outcomes were analyzed. A total of 68 patients were included, with a median age of 65.5 years (range: 60–79). The median preoperative Karnofsky performance scale (KPS) score was 90 (range 40–100) and median postoperative KPS score was 80 (range 0–90). Univariate analysis results showed that age, gender, comorbidities, preoperative KPS < 90 and MGMT promoter methylation were not significantly associated with PFS and OS. On the other hand, total resection, postoperative KPS ≥ 80, Ki67 > 25%, and Stupp-protocol treatment were significantly associated with prolonged PFS and OS. Moreover, multivariate analysis found that postoperative KPS ≥ 80, total resection, and Stupp-protocol treatment were prognostic factors for PFS and OS. The findings of this study have suggested that, on the premise of protecting function as much as possible, the more aggressive treatment regimens may prolong survival for elderly patients with GBM. However, further studies, particularly prospective randomized clinical trials, should be conducted to provide more definitive data on the appropriate management of elderly patients, especially for patients with MGMT promoter methylation.

## Introduction

Glioblastoma (GBM) is the most common malignant central nervous system (CNS) tumor (48.6%) and accounts for majority of gliomas (57.7%). It has been reported that its incidence rates increase with age and the median age at diagnosis is 65 years (Ostrom et al., 2020). Given the introduction of the “Stupp-protocol” in 2005 (Stupp et al., 2005), GBM patients have received standard treatment protocol. However, the study excluded patients aged over 70 years. Furthermore, in the 5-year analysis of the EORTC-NCIC trial (Stupp et al., 2009), patients older than 60 years had no survival advantage with combined therapy. This can be attributed to the poor tolerance and higher rates of adverse effects in elderly patients.

Generally, patients older than 65 or 70 years have been excluded in many clinical trials because previous studies identified age as an independent adverse prognostic factor in patients with GBM (Gulati et al., 2012; Raysi Dehcordi et al., 2012; Yang et al., 2013; Straube et al., 2020). Although several prospective trials have focused on de-escalated treatment of the elderly GBM patients, including radiotherapy or temozolomide monotherapy and short-course radiotherapy with temozolomide chemotherapy (Keime-Guibert et al., 2007; Malmström et al., 2012; Wick et al., 2012), none of these trials included standard concurrent chemoradiation over the course of 6 weeks as a control group. Moreover, the role of postoperative factors, especially postoperative Karnofsky performance scale (KPS), extent of resection (EOR), and standard concurrent chemoradiation, were still a matter of discussion. In this study, we performed a comprehensive analysis of elderly patients with glioblastoma treated at the Tangdu Hospital from 2015 to 2019, with the overarching goal of providing an overview of the influence of pre- and postoperative factors on progression-free and overall survival. Notably, all patients enrolled in the study were treated by a single medical team to ensure the homogeneity of surgery and therapeutic plan.

## Patients and Methods

This is a single-center retrospective clinical study performed at the Tangdu Hospital, Xian, China. We enrolled elderly glioblastoma patients (aged ≥ 60 years old at the time of operation) treated between January 2015 and December 2019. Patients were excluded from the study if they had recurrent lesions or received preoperative chemoradiotherapy.

The medical records of GBM patients were reviewed to obtain demographic information, such as age, gender, comorbidities, extent of surgery (partial or total resection), preoperative and postoperative KPS score, adjuvant treatment (Stupp-protocol or others) and the number of TMZ cycles. In addition, the patient pathology reports were reviewed to obtain molecular testing results, including O6-methylguanine-DNA methyltransferase (MGMT) promoter methylation status, isocitrate dehydrogenase (IDH) status, and Ki67 proliferation index (Ki67 index).

Preoperative and postoperative KPS were evaluated for all patients at admission and 1 week after surgery. The extent of resection was evaluated according to postoperative magnetic resonance (MR) images (within 72 h following surgery) by two experienced neuroradiologists. Total resection was defined as removal of 90–99% of the tumor mass, while partial tumor resection was defined as <90% resection (Orringer et al., 2012). Progression free survival (PFS) was defined as time from surgery to progression according to the RANO criteria (Wen et al., 2010). On the other hand, overall survival (OS) was defined as duration of time from surgical intervention until death or last follow-up.

All statistical analyses were performed using SPSS^®^ software (Version 20.0). Univariate survival analysis was performed using the Kaplan Meier method with the logrank test. All factors with a *p* < 0.10 on univariate analysis were included in the multivariable analyses. Multivariate survival analysis was performed using the Cox proportional-hazards regression model. The enumeration data were expressed as percentage and analyzed using the chi-square test. Notably, *p* < 0.05 was considered statistically significant.

The study protocol was approved by the local ethics committee of Tangdu Hospital.

## Results

### Overall Patient Characteristics

A total of 68 elderly patients with newly diagnosed GBM were initially included in this study. Table 1 shows the demographic data of the patients. Forty-one patients (60.3%) were male and the median age was 65.5 years (range 60–79). The ages of 17 patients (25.0%) were greater than or equal to 70 years. The most common comorbidities were hypertension (*n* = 20, 29.4%), followed by diabetes (*n* = 8, 11.8%), cardiovascular disease (*n* = 5, 7.4%), emphysema (*n* = 2, 2.9%), dilated cardiomyopathy (*n* = 1, 1.5%), sick sinus syndrome (*n* = 1, 1.5%), hypothyroidism (*n* = 1, 1.5%) and hyperthyroidism (*n* = 1, 1.5%). A total of 18 patients (26.5%) with one medical comorbidity and 9 patients (13.2%) had multiple comorbidities. Meanwhile, we reviewed the comorbidity of patients with different age groups (Age 60–65 and Age > 65). Based on our current study, there is no difference between two age groups (Supplementary Table 1). The median preoperative KPS was 90 (range 40–100), while the median postoperative KPS was 80 (range 0–90).

**TABLE 1:** Demographic date of all included patients.

Characteristics	*n* = 68
Age, years; median (range)	65.5 (60–79)
Sex, *n* (%)	
Male	41 (60.3%)
Female	27 (39.7%)
Comorbidities	
Hypertension	20 (29.4%)
Diabetes	8 (11.8%)
Cardiovascular disease	5 (7.4%)
Emphysema	2 (2.9%)
Dilated cardiomyopathy	1 (1.5%)
Sick sinus syndrome	1 (1.5%)
Hypothyroidism	1 (1.5%)
Hyperthyroidism	1 (1.5%)
With one comorbidity	18 (26.5%)
With multiple comorbidities	9 (13.2%)
Other cancer	2 (2.9%)
Tumor location, *n* (%)	
Temporal	31 (45.6%)
Frontal	25 (36.8%)
Parietal	18 (26.5%)
Occipital	14 (20.6%)
Insular	8 (11.8%)
Corpus callosum	1 (1.5%)
Cerebellum	1 (1.5%)
Preoperative KPS; median (range)	90 (40–100)
Postoperative KPS; median (range)	80 (0–90)
Surgery, *n* (%)	
Total resection	55 (80.9%)
Partial resection	13 (19.1%)
Postsurgical adjuvant treatment, *n* (%)	
Stupp-protocol	30 (47.1%)
Palliative treatment	35 (51.5%)
Others	3 (4.4%)
Ki67 index; median (range)	25% (5–80%)
MGMT promoter, *n* (%)	
Methylated	25 (48.1%)
Unmethylated	27 (51.9%)

*KPS, Karnofsky performance score; Ki67, index Ki67 proliferation index; MGMT, O6-methylguanine-DNA methyltransferase.*

All patients underwent surgical resection of lesions and total resection was achieved in 55 (80.9%) patients. After surgery, 30 patients (47.1%) were treated according to the “Stupp-protocol,” 35 patients (51.5%) did not receive any further treatment, and three patients (4.4%) received radiotherapy or chemotherapy alone. Furthermore, the median number of adjuvant TMZ cycles in our series was 10.5 (range, 1∼32) for patients who received “Stupp-protocol.” Eight patients (26.7%) received less than 6 cycles and twenty-two (73.3%) patients received more than 6 cycles. According to the preoperative magnetic resonance imaging, tumors were most often involved in the temporal lobe (*n* = 31), frontal lobe (*n* = 25), parietal (*n* = 18), and occipital lobe (*n* = 14). The tumors were less often in the insular lobe (*n* = 8), corpus callosum (*n* = 1), and cerebellum (*n* = 1).

MGMT promoter methylation status was available for 52 patients (76.5%), with 25 (48.1%) patients harboring MGMT promoter methylation. IDH status was known for 65 patients (95.6%) and only three patients (4.6%) were found to harbor IDH mutation. Moreover, Ki67 index was known for 62 patients (91.2%) with a median of 25% (range 5–80%).

The median follow up in the full cohort was 10.5 months (range 0.03–36 months). Results showed that the median PFS was 4.9 months, and the 1-and 2-year PFS rates were 26.5 and 3.4%, respectively. On the other hand, the median OS was 9.9 months, and the 1-and 2-year OS rates were 44.1 and 14.3%, respectively. It is worth noting that 61 patients (89.7%) had died at the time of analysis. One patient died due to perioperative complications (1.5%), while all other patients died due to disease recurrence.

Table 2 shows all the parameters tested for PFS and OS. Univariate analysis results revealed that age, gender, preoperative KPS, and MGMT promoter methylation were not significantly associated with PFS and OS. Further analysis indicated that either the presence of any comorbidity or the presence of multiple comorbidities had no significantly association with PFS and OS. IDH statistical analysis was not performed because of the small number of patients with IDH mutation.

**TABLE 2:** Significant parameters on PFS and OS (univariate analysis).

Characteristics	*N*	Median PFS (months)	*P*-value	Median OS (months)	*P*-value
Sex			0.796		0.808
Male	41	4.8		10.9	
Female	27	5.1		9.3	
Age			0.770		0.582
≤65 years	34	5.2		11.6	
66–70 years	17	4.9		10.9	
>70 years	17	3.6		9.0	
Comorbidity			0.429		0.862
None	41	5.1		7.9	
One	18	4.8		10.9	
Multiple	9	8.6		12.1	
Preoperative KPS			0.231		0.456
<90	30	4.8		7.7	
≥90	38	4.9		10.9	
Postoperative KPS			**<0.001**		**<0.001**
<80	31	3.5		5.3	
≥80	37	8.8		15.5	
Extent of resection			**0.002**		**0.008**
Total resection	55	5.9		5.5	
Partial resection	13	3.1		11.5	
Adjuvant treatment			**<0.001**		**<0.001**
Stupp	30	10.6		15.2	
Non- Stupp	38	3.5		5.3	
TMZ cycles			**<0.001**		**<0.001**
<6	8	3.6		7.8	
≥6	22	15.7		21.7	
MGMT promoter			0.838		0.845
Methylated	25	4.8		9.3	
Unmethylated	27	4.9		7.8	
Ki67 index			**0.027**		**0.026**
≤25%	35	8.4		12.8	
>25%	27	3.8		7.5	

*PFS, progression free survival; OS, overall survival; KPS, Karnofsky performance score; MGMT, O6-methylguanine-DNA methyltransferase; Ki67, index Ki67 proliferation index. Boldface type indicates statistical significance.*

**FIGURE 1:**
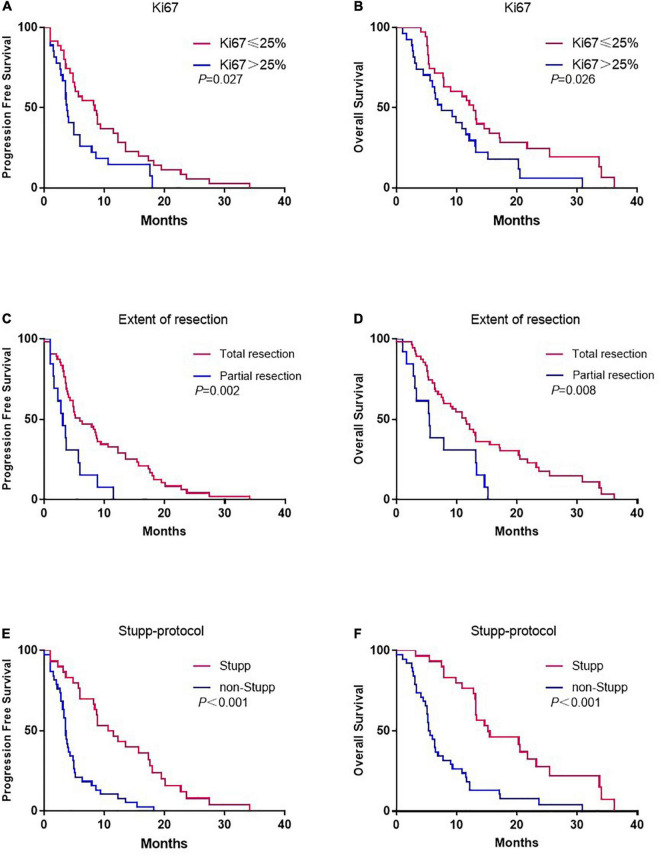
Kaplan-Meier estimates of progression-free survival and overall survival stratified by Ki67 **(A,B)**, extent of resection **(C,D)**, and Stupp-protocol **(E,F)**.

According to the Ki67 index, patients with Ki67 ≤ 25% were associated with significantly prolonged PFS (8.4 vs. 3.8 months, *p* = 0.027) (Figure 1A) and OS (12.8 vs. 7.5 months, *p* = 0.026) (Figure 1B). According to the extent of surgery, total resection significantly prolonged PFS (5.9 vs. 3.1 months, *p* = 0.002) (Figure 1C) and OS (11.5 vs. 5.5 months, *p* = 0.008) (Figure 1D) compared to partial resection. The same pattern was observed with adjuvant treatment, with longer PFS (10.6 vs. 3.6 months, *p* < 0.001) (Figure 1E) and OS (15.2 vs. 5.6 months, *p* < 0.001) (Figure 1F) in the group that received Stupp-protocol compared to those that did not.

**FIGURE 2:**
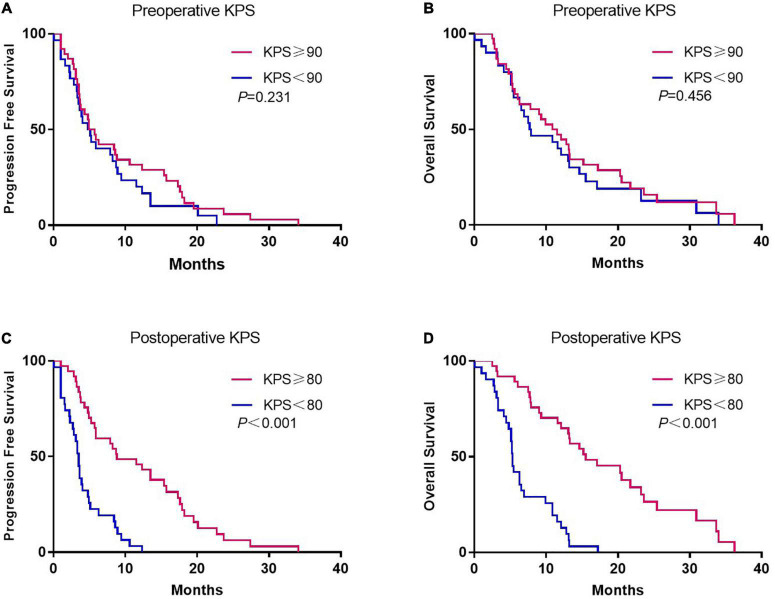
Kaplan-Meier curves of progression-free survival and overall survival according to preoperative **(A,B)** and postoperative KPS **(C,D)**.

**FIGURE 3:**
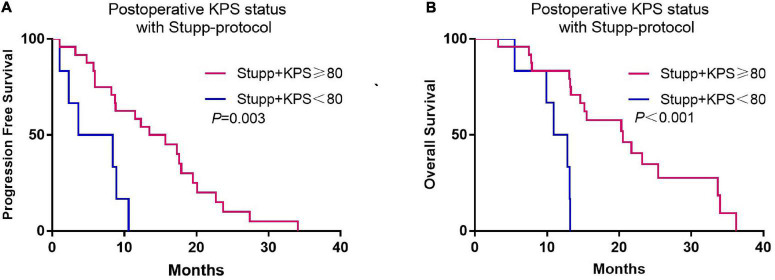
Kaplan-Meier curves of progression-free survival **(A)** and overall survival **(B)** according to postoperative KPS status (KPS ≥ 80 and KPS < 80) for patients who received standard Stupp-protocol.

Although there was no association between preoperative KPS and survival, postoperative KPS exhibited significant differences (Figure 2). The PFS (8.8 vs. 3.5 months, *p* < 0.001) and OS (15.5 vs. 5.3 months, *p* < 0.001) were significantly longer in patients with postoperative KPS ≥ 80. Notably, more patients with KPS ≥ 80 received Stupp-protocol treatment compared to patients with KPS<80 (64.9% vs. 22.6% *p* = 0.001). The prognostic effect was further analyzed in patients who received Stupp-protocol treatment. It is worth noting that postoperative KPS ≥ 80 remained the significant prognostic factor for patients who received standard Stupp regimen (Figure 3). Patients who received adjuvant TMZ for more than 6 cycles had longer PFS (15.7 vs. 3.6 months, *p <* 0.001) and OS (21.7 vs. 7.8 months, *p* < 0.001) than patients who received fewer than 6 cycles.

Furthermore, multivariate analysis showed that postoperative KPS, total resection, and Stupp-protocol treatment were prognostic factors for PFS and OS. Ki67 ≤ 25% showed a non-significant influence on PFS (*p* = 0.051) and OS (*p* = 0.076) (Table 3).

**TABLE 3:** Cox proportional hazards model for OS in all patients.

Parameter	*P*-value	HR	95% CI of HR	
			Lower	Upper
Postoperative KPS	**<0.001**	0.263	0.136	0.511
Extent of resection	**<0.001**	0.234	0.113	0.486
Adjuvant treatment	**<0.001**	0.272	0.137	0.541
Ki67 index	0.079	0.592	0.331	1.062

*OS, overall survival; KPS, Karnofsky performance score; Ki67, index Ki67 proliferation index; HR, hazard ratio; CI, confidence interval. Boldface type indicates statistical significance.*

## Discussion

The CBTRUS statistical report indicated that glioblastoma is the most common of all malignant CNS tumors in adults, its incidence rates increase with advancing age, and the median age at diagnosis is 65 years (Ostrom et al., 2020). Management of elderly patients with GBM is difficult due to the poor prognosis, multiple comorbidities, and an increased risk of adverse effects from radiotherapy (Perry et al., 2017). Therefore, most clinical trials have excluded patients older than 65 years, which has resulted in no uniform optimal chemotherapy regimen and treatment protocol for elderly patients with GBM (Stupp et al., 2005; Palmer et al., 2018). Herein, we present a retrospective analysis of elderly patients with GBM who were treated by a single medical team, which suggests that this study has higher concordance in surgery and therapeutic plan.

The KPS allows patients to be classified according to their performance status. Previous studies reported that the preoperative KPS scores are predictors of outcome in patients with glioblastoma (Chaichana et al., 2011a,^2013^; Marina et al., 2011). In this study, it was found that the preoperative KPS scores had no significant correlation with PFS and OS. In contrast, the postoperative KPS score was associated with increased survival in univariate analyses. Similarly, Chambless et al. (2015) conducted a retrospective review of 161 glioblastoma patients (mean age 61 ± 15 years) and found that the postoperative KPS was associated with prolonged OS, but preoperative KPS was not. In addition, Pontes Lde et al. (2013) found that postoperative KPS ≥ 70 was associated with longer OS, but the study did not determine the impact of preoperative KPS on survival. To the best of our knowledge, this is the first study to demonstrate that postoperative KPS score has superior predictive value compared to preoperative KPS score in elderly patients with glioblastoma. Notably, most of the patients in the sub-group of patients with higher postoperative KPS score received aggressive therapy, which could be one explanation for this finding.

Evidence have suggested that elderly patients have higher incidence rates of medical comorbidities. However, it is still unclear on the relationship between comorbidities and survival in elderly patients with GBM. Previous study shown that the patients with any or multiple comorbidities had similar survival to patients without medical comorbidities (Voisin et al., 2021). In our current series, we did not find the presence of medical comorbidities significantly correlated with patients’ survival. This was consistent with the report. Meanwhile, Montemurro et al. (2020) investigated the influence of diabetes, hyperglycemia and metformin on OS of patients with GBM. They found that the hyperglycemia, rather than diabetes was an independent risk factor for poor outcome and shorter OS in patients with GBM. One retrospective study found long-term lower systolic blood pressure, higher blood glucose and lower serum albumin level were associated with shorter survival in GBM patients (Liu et al., 2019). Whereas it is noteworthy that the patients analyzed in these studies were not only elderly patients but all patients with GBM. Therefore, further research was needed for better understanding the relationship between medical comorbidities and survival in elderly patients with GBM.

Surgery is the primary treatment for glioblastoma. Most studies have proposed that the extent of the initial surgical resection is an important prognostic factor (Martinez et al., 2007; Chaichana et al., 2011b; Malmström et al., 2012; Li et al., 2017). The goals of surgery are maximal safe tumor resection, and obtaining clinicopathological and molecular genetic results. A meta-analysis which included six articles involving 1,618 glioblastoma patients showed that total resection is associated with improved OS and PFS compared to incomplete resection and biopsy (Li et al., 2017). Moreover, several retrospective reviews have revealed that gross-total resection confers a significant survival benefit on elderly patients with glioblastoma and without increased surgery-related morbidity (Chaichana et al., 2011b). Similarly, a recent study demonstrated that aggressive surgery is technically feasible in elderly patients (Barbagallo et al., 2020). Therefore, total resection is feasible in elderly patients with glioblastoma because it is safe and efficacious. Likewise, this study showed that patients who underwent total resection were well-tolerated and had significantly prolonged survival.

It should be noted that there is no standard definition for “elderly” patients (Zarnett et al., 2015). Some studies have defined elderly patients as patients aged above 65 years (Hoffermann et al., 2015; Youssef et al., 2019). The “NOA-08” (Wick et al., 2012) and “Nordic” (Malmström et al., 2012) trials defined elderly patients as being 65 and 60 years, respectively. In China, age of 60 years old has been defined as elderly for a long time and most people retire at the age of 60 years. Therefore, this study used age of 60 years old as the age cut-off value. Most previous studies on glioblastoma have reported that age is one of the most important factors which influence OS in elderly patients (Laws et al., 2003; Lamborn et al., 2004; Stupp et al., 2009). On the other hand, some studies have suggested that there is no significant correlation between age and survival time. According to Oszvald et al. (2012), the OS of elderly patients was significantly lower than that of younger patients, but when they stratified between resection and biopsy, age was not a negative prognostic factor in patients undergoing complete tumor resection. A retrospective chart review that included elderly GBM patients found that higher KPS and chemoradiotherapy were independently associated with improved OS, but age was not (Youssef et al., 2019). This study found no difference in the survival of patients in different age cohorts (ages 60–65 vs. 66–70 vs. 71 and older). Therefore, these findings suggest that age should not be used as the basis for treatment decisions or as an exclusion criterion in clinical trials.

Considering that temozolomide has become the standard treatment for glioblastoma, it is very important to evaluate the therapeutic effect of temozolomide in elderly glioblastoma patients. In 2009, the 5-year survival analysis of the EORTC-NCIC trial showed that the survival advantage was less pronounced among patients aged between 60 and 70 years (Stupp et al., 2009). Given the poor prognosis in elderly patients, many studies have focused on determining whether a shorter course of radiotherapy could replace standard radiotherapy. Roa et al. (2004) reported that there was no difference in survival between patients receiving standard RT (60 Gy in 30 fractions) or short-course RT (40 Gy in 15 fractions) in patients aged 60 years or older. The Nordic trial randomized glioblastoma patients aged 60 years or older into three groups: temozolomide, hypofractionated radiotherapy, or standard radiotherapy. The study found that standard radiotherapy was associated with the poorest outcomes, especially in patients older than 70 years (Malmström et al., 2012). Furthermore, a recent systematic review and network meta-analysis found that there was a trend toward improved survival with combined therapies (radiotherapy with temozolomide) compared to single modality therapies (either radiotherapy or chemotherapy alone) (Nassiri et al., 2020). Herein, the median OS of patients who received concurrent chemoradiation and adjuvant temozolomide was 15.2 months, which was consistent with the OS reported by the EORTC-NCIC trial (median OS: 14.6 months).

There are controversies on the optimal cycles of adjuvant temozolomide in patients with GBM. A retrospective study implied that extended adjuvant TMZ was safe and may prolong survival in patients with GBM (Roldán Urgoiti et al., 2012). Similarly, Lwin et al. (2013) in a retrospective study of 433 patients with GBM, found that longer cycles of TMZ chemotherapy was associated with longer survival in patients with GBM. These results are consistent with the findings of our study. Conversely, the opposite view also exists. A meta-analysis pooled 4 randomized controlled trials (RCT) of patients with newly diagnosed GBM suggested that adjuvant TMZ beyond 6 cycles did not improve OS, even for patients with MGMT promoter methylation (Blumenthal et al., 2017). A recent prospective, phase 2 study showed that extended adjuvant TMZ did not improve PFS or OS, which was linked to the increased toxicity (Balana et al., 2020). However, it should be noted that the enrolled patients in their study had completed six cycles of TMZ chemotherapy and without progression. In our current study, the main reason for patients who received less than 6 cycles of TMZ chemotherapy was disease progression, which was likely the reason why those patients had poor survival.

All of the previous trials have demonstrated that MGMT promoter methylation is a biomarker of outcome, and is a strong predictor of benefit with temozolomide chemotherapy (Esteller et al., 2000). In the NOA-08 trial, the MGMT promoter methylation was observed in 73 (35%) of 209 patients and was associated with longer OS (Wick et al., 2012). In addition, the Nordic trial found that patients who had MGMT promoter methylation had better survival after temozolomide treatment than those without MGMT promoter methylation (Malmström et al., 2012). In this study, it was found that the median survival for patients who received standard Stupp regimen was 20.5 months for methylated and 13.2 months for non-methylated cases (*P* = 0.370). However, the difference was not statistically significant probably due to the small number of patients.

The EF-14 phase 3 study showed that, compared with TMZ treatment alone, TTFields plus TMZ significantly prolonged survival in patients with newly diagnosed glioblastoma (Stupp et al., 2017). The role of the TTFields in elderly patients with GBM was also investigated in the subgroup analysis of EF-14 trial, which found that the TTFields and TMZ combination treatment can significantly prolonged the PFS (6.5 vs. 3.9 months, *P* = 0.0236) and OS (17.4 vs. 13.7 months, *P* = 0.0204) compared to TMZ alone (Ram et al., 2021). Moreover, this study and recent global post-marketing safety surveillance analysis demonstrated the tolerability and safety of TTFields for elderly patients with GBM (Shi et al., 2020; Ram et al., 2021), further analysis found that the most common TTFields-related adverse event is mild-to-moderate skin reactions with a manageable toxicity profile. In our study, two patients received TMZ chemotherapy combined with TTFields have longer survival and still in follow-up (23.8 and 30.3 months after first diagnosis and surgical intervention, respectively). Therefore, we suggest that TTFields and TMZ combination therapy is effective and relatively safe for elderly patients with GBM.

## Limitation

Although the findings of this study are encouraging, it had some limitations. First, this is a retrospective review at a single institution which has its inherent limitations. Second, despite the fact that all of patients were treated by a single medical team, the number of patients included in this study was small and each treatment protocol has certain inconsistencies. Third, several important molecular markers such as TERT promoter mutation and ATRX mutation, were not available. Fourth, the adverse effects were not analyzed and therapies for recurrent cases were not considered.

## Conclusion

This study has shown that total resection, aggressive treatment, and postoperative KPS score were associated with improved survival of elderly GBM patients. The results have suggested that, on the premise of protecting function as much as possible, the most suitable treatment strategies for elderly patients with GBM should be maximal safe resection combined with adjuvant chemoradiotherapy. However, further studies, particularly prospective randomized clinical trials, should be conducted to provide more definitive data on the appropriate management of elderly patients, especially for patients with MGMT promoter methylation.

## Data Availability Statement

The original contributions presented in the study are included in the article/Supplementary Material, further inquiries can be directed to the corresponding author/s.

## Ethics Statement

Ethical review and approval was not required for the study on human participants in accordance with the local legislation and institutional requirements. Written informed consent for participation was not required for this study in accordance with the national legislation and the institutional requirements.

## Author Contributions

JL performed most of the data analyses and drafted the manuscript. CL performed most of the clinical analyses (imaging data) together with YW and contributed to the writing of the manuscript. PJ contributed to the data analyses together with SCG, YZ, YJ, and WZ. NW performed most of the clinical follow-up with MX, MC, and FF. LW performed the clinical analyses and designed the study together with JL, SNG, and YQ. All authors contributed to the article and approved the submitted version.

## Conflict of Interest

The authors declare that the research was conducted in the absence of any commercial or financial relationships that could be construed as a potential conflict of interest.

## Publisher’s Note

All claims expressed in this article are solely those of the authors and do not necessarily represent those of their affiliated organizations, or those of the publisher, the editors and the reviewers. Any product that may be evaluated in this article, or claim that may be made by its manufacturer, is not guaranteed or endorsed by the publisher.

## References

[B1] Balana C. Vaz M. A. Manuel Sepúlveda J. Mesia C. Del Barco S. Pineda E. (2020). A phase II randomized, multicenter, open-label trial of continuing adjuvant temozolomide beyond 6 cycles in patients with glioblastoma (GEINO 14-01). *Neuro Oncol.* 22 1851–1861. 10.1093/neuonc/noaa107 32328662 PMC7746946

[B2] Barbagallo G. M. V. Altieri R. Garozzo M. Maione M. Di Gregorio S. Visocchi M. (2020). High Grade Glioma treatment in elderly people: is it different than in younger patients? Analysis of surgical management guided by an intraoperative multimodal approach and its impact on clinical outcome. *Front. Oncol.* 10:631255. 10.3389/fonc.2020.631255 33718122 PMC7943843

[B3] Blumenthal D. T. Gorlia T. Gilbert M. R. Kim M. M. Burt Nabors L. Mason W. P. (2017). Is more better? The impact of extended adjuvant temozolomide in newly diagnosed glioblastoma: a secondary analysis of EORTC and NRG Oncology/RTOG. *Neuro Oncol.* 19 1119–1126. 10.1093/neuonc/nox025 28371907 PMC5570239

[B4] Chaichana K. L. Chaichana K. K. Olivi A. Weingart J. D. Bennett R. Brem H. (2011a). Surgical outcomes for older patients with glioblastoma multiforme: preoperative factors associated with decreased survival. *J. Neurosurg.* 114 587–594. 10.3171/2010.8.JNS1081 20887095 PMC4020429

[B5] Chaichana K. L. Garzon-Muvdi T. Parker S. Weingart J. D. Olivi A. Bennett R. (2011b). Supratentorial glioblastoma multiforme: the role of surgical resection versus biopsy among older patients. *Ann. Surg. Oncol.* 18 239–245. 10.1245/s10434-010-1242-6 20697823 PMC4612568

[B6] Chaichana K. L. Martinez-Gutierrez J. C. De la Garza-Ramos R. Weingart J. D. Olivi A. Gallia G. L. (2013). Factors associated with survival for patients with glioblastoma with poor pre-operative functional status. *J. Clin. Neurosci.* 20 818–823. 10.1016/j.jocn.2012.07.016 23639620 PMC3994533

[B7] Chambless L. B. Kistka H. M. Parker S. L. Hassam-Malani L. McGirt M. J. Thompson R. C. (2015). The relative value of postoperative versus preoperative Karnofsky Performance Scale scores as a predictor of survival after surgical resection of glioblastoma multiforme. *J. Neurooncol.* 121 359–364. 10.1007/s11060-014-1640-x 25344883

[B8] Esteller M. Garcia-Foncillas J. Andion E. Goodman S. N. Hidalgo O. F. Vanaclocha V. (2000). Inactivation of the DNA-repair gene MGMT and the clinical response of gliomas to alkylating agents. *N. Engl. J. Med.* 343 1350–1354. 10.1056/NEJM200011093431901 11070098

[B9] Gulati S. Jakola A. S. Johannesen T. B. Solheim O. (2012). Survival and treatment patterns of glioblastoma in the elderly: a population-based study. *World Neurosurg.* 78 518–526. 10.1016/j.wneu.2011.12.008 22381305

[B10] Hoffermann M. Bruckmann L. Kariem Mahdy A. Asslaber M. Payer F. von Campe G. (2015). Treatment results and outcome in elderly patients with glioblastoma multiforme–a retrospective single institution analysis. *Clin. Neurol. Neurosurg.* 128 60–69. 10.1016/j.clineuro.2014.11.006 25462098

[B11] Keime-Guibert F. Chinot O. Taillandier L. Cartalat-Carel S. Frenay M. Kantor G. (2007). Radiotherapy for glioblastoma in the elderly. *N. Engl. J. Med.* 356 1527–1535. 10.1056/NEJMoa065901 17429084

[B12] Lamborn K. R. Chang S. M. Prados M. D. (2004). Prognostic factors for survival of patients with glioblastoma: recursive partitioning analysis. *Neuro Oncol.* 6 227–235. 10.1215/S1152851703000620 15279715 PMC1871999

[B13] Laws E. R. Parney I. F. Huang W. Anderson F. Morris A. M. Asher A. (2003). Survival following surgery and prognostic factors for recently diagnosed malignant glioma: data from the Glioma Outcomes Project. *J. Neurosurg.* 99 467–473. 10.3171/jns.2003.99.3.0467 12959431

[B14] Li X. Z. Li Y. B. Cao Y. Li P. L. Liang B. Sun J. D. (2017). Prognostic implications of resection extent for patients with glioblastoma multiforme: a meta-analysis. *J. Neurosurg. Sci.* 61 631–639. 10.23736/S0390-5616.16.03619-5 26824196

[B15] Liu W. Qdaisat A. Yeung J. Lopez G. Weinberg J. Zhou S. (2019). The Association Between Common Clinical Characteristics and Postoperative Morbidity and Overall Survival in Patients with Glioblastoma. *Oncologist* 24 529–536. 10.1634/theoncologist.2018-0056 30049883 PMC6459250

[B16] Lwin Z. MacFadden D. Al-Zahrani A. Atenafu E. Miller B. A. Sahgal A. (2013). Glioblastoma management in the temozolomide era: Have we improved outcome? *J. Neurooncol.* 115 303–310. 10.1007/s11060-013-1230-3 23979682

[B17] Malmström A. Grønberg B. H. Marosi C. Stupp R. Frappaz D. Schultz H. (2012). Temozolomide versus standard 6-week radiotherapy versus hypofractionated radiotherapy in patients older than 60 years with glioblastoma: the Nordic randomised, phase 3 trial. *Lancet Oncol.* 13 916–926. 10.1016/S1470-2045(12)70265-6 22877848

[B18] Marina O. Suh J. H. Reddy C. A. Barnett G. H. Vogelbaum M. A. Peereboom D. M. (2011). Treatment outcomes for patients with glioblastoma multiforme and a low Karnofsky Performance Scale score on presentation to a tertiary care institution. *J. Neurosurg.* 115 220–229. 10.3171/2011.3.JNS10495 21548745

[B19] Martinez R. Janka M. Soldner F. Behr R. (2007). Gross-total resection of malignant gliomas in elderly patients: implications in survival. *Zentralbl. Neurochir.* 68 176–181. 10.1055/s-2007-985851 17963194

[B20] Montemurro N. Perrini P. Rapone B. (2020). Clinical Risk and Overall Survival in Patients with Diabetes Mellitus, Hyperglycemia and Glioblastoma Multiforme. A Review of the Current Literature. *Int. J. Environ. Res. Public Health* 17:8501. 10.3390/ijerph17228501 33212778 PMC7698156

[B21] Nassiri F. Taslimi S. Wang J. Z. Badhiwala J. H. Dalcourt T. Ijad N. (2020). Determining the optimal adjuvant therapy for improving survival in elderly patients with glioblastoma: a systematic review and network meta-analysis. *Clin. Cancer Res.* 26 2664–2672. 10.1158/1078-0432.CCR-19-3359 31953312

[B22] Orringer D. Lau D. Khatri S. Zamora-Berridi G. J. Zhang K. Wu C. (2012). Extent of resection in patients with glioblastoma: limiting factors, perception of resectability, and effect on survival. *J. Neurosurg.* 117 851–859. 10.3171/2012.8.JNS12234 22978537

[B23] Ostrom Q. T. Patil N. Cioffi G. Waite K. Kruchko C. Barnholtz-Sloan J. S. (2020). CBTRUS Statistical Report: primary brain and other central nervous system Tumors Diagnosed in the United States in 2013-2017. *Neuro Oncol.* 22(12 Suppl. 2), iv1–iv96. 10.1093/neuonc/noaa200 33123732 PMC7596247

[B24] Oszvald A. Güresir E. Setzer M. Vatter H. Senft C. Seifert V. (2012). Glioblastoma therapy in the elderly and the importance of the extent of resection regardless of age. *J. Neurosurg.* 116 357–364. 10.3171/2011.8.JNS102114 21942727

[B25] Palmer J. D. Bhamidipati D. Mehta M. Williams N. L. Dicker A. P. Werner-Wasik M. (2018). Treatment recommendations for elderly patients with newly diagnosed glioblastoma lack worldwide consensus. *J. Neurooncol.* 140 421–426. 10.1007/s11060-018-2969-3 30088191

[B26] Perry J. R. Laperriere N. O’Callaghan C. J. Brandes A. A. Menten J. Phillips C. (2017). Short-Course Radiation plus Temozolomide in Elderly Patients with Glioblastoma. *N. Engl. J. Med.* 376 1027–1037. 10.1056/NEJMoa1611977 28296618

[B27] Pontes Lde B. Loureiro L. V. Koch L. O. Karnakis T. Guendelmann R. A. Weltman E. (2013). Patterns of care and outcomes in elderly patients with glioblastoma in Sao Paulo, Brazil: a retrospective study. *J. Geriatr. Oncol.* 4 388–393. 10.1016/j.jgo.2013.07.005 24472484

[B28] Ram Z. Kim C. Y. Hottinger A. F. Idbaih A. Nicholas G. Zhu J. J. (2021). Efficacy and Safety of Tumor Treating Fields (TTFields) in Elderly Patients with Newly Diagnosed Glioblastoma: subgroup analysis of the phase 3 EF-14 Clinical Trial. *Front. Oncol.* 11:671972. 10.3389/fonc.2021.671972 34692470 PMC8526342

[B29] Raysi Dehcordi S. De Paulis D. Marzi S. Ricci A. Cimini A. Cifone M. G. (2012). Survival prognostic factors in patients with glioblastoma: our experience. *J. Neurosurg. Sci.* 56 239–245. 22854592

[B30] Roa W. Brasher P. M. Bauman G. Anthes M. Bruera E. Chan A. (2004). Abbreviated course of radiation therapy in older patients with glioblastoma multiforme: a prospective randomized clinical trial. *J. Clin. Oncol.* 22 1583–1588. 10.1200/JCO.2004.06.082 15051755

[B31] Roldán Urgoiti G. B. Singh A. D. Easaw J. C. (2012). Extended adjuvant temozolomide for treatment of newly diagnosed glioblastoma multiforme. *J. Neurooncol.* 108 173–177. 10.1007/s11060-012-0826-3 22382781

[B32] Shi W. Blumenthal D. T. Oberheim Bush N. A. Kebir S. Lukas R. V. Muragaki Y. (2020). Global post-marketing safety surveillance of Tumor Treating Fields (TTFields) in patients with high-grade glioma in clinical practice. *J. Neurooncol.* 148 489–500. 10.1007/s11060-020-03540-6 32535723 PMC7438370

[B33] Straube C. Kessel K. A. Antoni S. Gempt J. Meyer B. Schlegel J. (2020). A balanced score to predict survival of elderly patients newly diagnosed with glioblastoma. *Radiat. Oncol.* 15:97. 10.1186/s13014-020-01549-9 32375830 PMC7201994

[B34] Stupp R. Hegi M. E. Mason W. P. van den Bent M. J. Taphoorn M. J. Janzer R. C. (2009). Effects of radiotherapy with concomitant and adjuvant temozolomide versus radiotherapy alone on survival in glioblastoma in a randomised phase III study: 5-year analysis of the EORTC-NCIC trial. *Lancet Oncol.* 10 459–466. 10.1016/S1470-2045(09)70025-7 19269895

[B35] Stupp R. Mason W. P. van den Bent M. J. Weller M. Fisher B. Taphoorn M. J. (2005). Radiotherapy plus concomitant and adjuvant temozolomide for glioblastoma. *N. Engl. J. Med.* 352 987–996. 10.1056/NEJMoa043330 15758009

[B36] Stupp R. Taillibert S. Kanner A. Read W. Steinberg D. Lhermitte B. (2017). Effect of Tumor-Treating Fields Plus maintenance temozolomide vs maintenance temozolomide alone on survival in patients with glioblastoma: a randomized clinical trial. *JAMA* 318 2306–2316. 10.1001/jama.2017.18718 29260225 PMC5820703

[B37] Voisin M. R. Sasikumar S. Zadeh G. (2021). Predictors of survival in elderly patients undergoing surgery for glioblastoma. *Neurooncol. Adv.* 3:vdab083. 10.1093/noajnl/vdab083 34355171 PMC8331047

[B38] Wen P. Y. Macdonald D. R. Reardon D. A. Cloughesy T. F. Sorensen A. G. Galanis E. (2010). Updated response assessment criteria for high-grade gliomas: response assessment in neuro-oncology working group. *J. Clin. Oncol.* 28 1963–1972. 10.1200/JCO.2009.26.3541 20231676

[B39] Wick W. Platten M. Meisner C. Felsberg J. Tabatabai G. Simon M. (2012). Temozolomide chemotherapy alone versus radiotherapy alone for malignant astrocytoma in the elderly: the NOA-08 randomised, phase 3 trial. *Lancet Oncol.* 13 707–715. 10.1016/S1470-2045(12)70164-X 22578793

[B40] Yang P. Wang Y. Peng X. You G. Zhang W. Yan W. (2013). Management and survival rates in patients with glioma in China (2004-2010): a retrospective study from a single-institution. *J. Neurooncol.* 113 259–266. 10.1007/s11060-013-1103-9 23483435

[B41] Youssef M. Ludmir E. B. Mandel J. J. Patel A. J. Jalali A. Treiber J. (2019). Treatment strategies for glioblastoma in older patients: age is just a number. *J. Neurooncol.* 145 357–364. 10.1007/s11060-019-03304-x 31643011

[B42] Zarnett O. J. Sahgal A. Gosio J. Perry J. Berger M. S. Chang S. (2015). Treatment of elderly patients with glioblastoma: a systematic evidence-based analysis. *JAMA Neurol.* 72 589–596. 10.1001/jamaneurol.2014.3739 25822375

