# Supplementary material for: Prognostic and Predictive Factors in Elderly Patients With Glioblastoma: A Single-Center Retrospective Study
Source: Front Aging Neurosci. 2022 Jan 31;13:777962. doi: 10.3389/fnagi.2021.777962 (PMC8841486; doi:10.3389/fnagi.2021.777962)
Supplement: Supplementary file 1 [file Table_1.DOCX]

Supplementary Table Patients comorbidities in accordance with age

| Comorbidities | Cohort | | | *P* value |
| --- | --- | --- | --- | --- |
|  | All (68) | age 60-65 (34) | age＞65 (34) |  |
| Hypertension | 20 (29.4%) | 9 (26.5%) | 11 (32.4%) | 0.5945 |
| Diabetes | 8 (11.8%) | 2 (5.9%) | 6 (17.6%) | 0.2585 |
| Cardiovascular disease | 5 (7.4%) | 1 (2.9%) | 4 (11.8) | 0.3559 |
| Emphysema | 2 (2.9%) | 0 | 2 (5.9%) | 0.4925 |
| Dilated cardiomyopathy | 1 (1.5%) | 1 (2.9%) | 0 | >0.9999 |
| Sick sinus syndrome | 1 (1.5%) | 1 (2.9%) | 0 | >0.9999 |
| Hypothyroidism | 1 (1.5%) | 1 (2.9%) | 0 | >0.9999 |
| Hyperthyroidism | 1 (1.5%) | 1 (2.9%) | 0 | >0.9999 |
| With any comorbidities | 27 (39.7%) | 12 (35.3%) | 15 (44.1%) | 0.4572 |
| With multiple comorbidities | 9 (13.2%) | 3 (8.8%) | 6 (17.6%) | 0.4763 |
